# The inflammation saga: Breakthrough nutritional insights for poultry

**DOI:** 10.1016/j.psj.2025.106200

**Published:** 2025-12-05

**Authors:** Rajesh Jha, Doug Korver, Woo Kyun Kim, Leon Marchal, Kirsty Gibbs, John Halley

**Affiliations:** a University of Hawaii at Manoa, Honolulu, HI, USA; b Alpine Poultry Nutrition, Inc., Edmonton, AB, Canada; c University of Alberta, Edmonton, AB, Canada; d University of Georgia, Athens, GA, USA; e Danisco Animal Nutrition & Health, IFF, Oegstgeest, the Netherlands; f J. Halley Poultry Consulting LLC, Siloam Springs, AR, USA

**Keywords:** Gut Inflammation, Nutrition, Fiber, Amino acids, Mineral chelation

## Abstract

This symposium offered a broader perspective on gut inflammation in poultry. It covered the basic factors that trigger gut inflammation, their potential effects on performance and health, and potential strategies to manage inflammation and mitigate its adverse effects. Gut inflammation has emerged as a critical determinant of health and performance in modern poultry production. A focus was placed on key nutritional factors that influence gut integrity and immune activation, including the pro- and anti-inflammatory roles of specific feed components. The role of dietary fiber was discussed, emphasizing its dualistic effects as both a potential irritant and a modulator of microbial populations and immune functions. The symposium also discussed the roles of amino acids such as threonine, glutamine, and arginine, which aid in mucosal repair and immune modulation. Additionally, the impact of mineral chelation, particularly involving zinc and copper, was discussed in the context of enhancing mineral bioavailability while minimizing oxidative and inflammatory responses. Ultimately, the symposium explored a practical approach to managing gut inflammation in commercial poultry operations, emphasizing the importance of early nutritional interventions and targeted feed formulation and nutrition strategies. Lastly, it was concluded that by integrating current scientific evidence with practical insights, poultry nutritionists and industry stakeholders should employ multiple approaches based on actionable knowledge to optimize gut health and reduce inflammation, thereby enhancing bird performance and the sustainability of the poultry industry.

## Nutritional factors of gut inflammation (D. Korver)

The intestinal tract is one of the largest, if not the largest, surface areas exposed to the environment of humans and poultry (Ferrer et al., 2003; Helander and Fandriks, 2014). Although the digestive tract is contained within the body, the lumen is actually outside of the bird. Therefore, although the function of the intestinal tract is usually thought of as being the uptake of nutrients, it has another important function: preventing access of microbes and toxins to the tissues of the bird (Ducatelle et al., 2023). The intestinal tract has multiple layers of defense, including passive mechanisms such as mucin, tight junction proteins, and the epithelial surface. Additionally, the immune system has a large presence in the intestinal tract, monitoring the presence of commensal and pathogenic microorganisms, and detecting proliferation of potential pathogens within the gut.

Inflammation is a biological reaction to disrupted tissue homeostasis (Medzhitov, 2008) intended to rapidly destroy or isolate the cause of the disruption, and to remove damaged tissue and restore homeostasis. It is a tissue-destroying process that not only disrupts invading pathogens but can also destroy or impair the function of the host’s own tissues (Ashley et al., 2012). Inflammation is a critical part of the host’s protection from disease. However, it can become harmful to the host when the inflammation becomes too intense (e.g., “cytokine storm”) or when the inflammation becomes chronic and is not resolved (Oronsky et al., 2022). Intestinal dysbiosis refers to an imbalance in the function and composition of the intestinal microflora. Situations in which certain microbial groups are favored while others are suppressed can lead to changes in intestinal morphology and function. This change in microbiota and translocation of microbes leads to localized inflammation and oxidative stress, which decreases gut barrier and absorptive functions, and increases the availability of nutrients to the intestinal microbes (Fathima et al., 2022; Ducatelle et al., 2023). This oversupply of nutrients in the lumen of the gut further disrupts the balance of microbes, leading to further inflammation and oxidation, creating a cycle of disruption (De Gussem, 2010).

If a microbe is able to breach the passive intestinal defenses including the mucus layer and tight junction proteins, localized inflammatory responses are initiated to limit the translocation of the pathogen. Cells of the adaptive immune response are recruited and activated, and if a more vigorous response is necessary, the inflammation can become systemic. The activation of a systemic inflammatory response reduces poultry performance through a number of different mechanisms. The primary effect, particularly in young birds, is a dramatic reduction in feed intake, thus reducing the supply of building blocks for growth. This reduction in feed intake accounts for about 71 % of the lost performance associated with strong systemic inflammation (Klasing, 2017). However, additional mechanisms such as the cost of increasing protective barriers at the level of the gut (5 % of lost performance), a mobilization of muscle protein to support protective acute phase protein production (8 %), fever (3.5 %), and reduced efficiency of nutrient absorption (9 %) also contribute to the loss of performance (Klasing, 2017).

The use of antibiotic growth promoters (AGP) allowed growing birds to achieve a greater proportion of their genetic potential for growth. In a meta-analysis of 174 peer-reviewed research studies, reporting 183 individual experiments, the loss in performance due to the absence of AGP was evaluated (Cardinal et al., 2019). Feed conversion ratio from day 1-42 increased from 1.66 to 1.72, and the cost of production increased by $0.03 USD per bird, resulting in an estimated economic impact of $183,560,232 (USD) per year when extrapolated to the size of the Brazilian broiler industry at the time. Although AGP are commonly assumed to function indirectly by reducing inflammation through the control of gut microbial activity (summarized by Plata et al., 2022), evidence suggests that AGP may also exert a direct effect on immune cells, further reducing inflammation (Niewold, 2007). Regardless of the mechanism(s) of action, the global move away from AGP requires both an understanding of the nature of intestinal inflammation and the development of effective alternatives to replace AGP in poultry diets.

The impact of systemic inflammation is observed even when clinical disease is absent. In 7-week-old cross-bred chickens housed in cages, the birds having the highest body weight had lower levels of serum lipopolysaccharide (indicating increased intestinal barrier function) and markers of systemic inflammation, and increased intestinal barrier function markers (Zhang et al., 2022). In a challenge model intended to identify biomarkers for the breakdown of the host intestinal barrier function, either a corn-soy or a rye-wheat-barley (challenge) grower diet without supplemental enzymes was fed to broilers. In addition, the challenge birds were gavaged with a 2X oral coccidiosis vaccine at day 21. The challenged birds had reduced body weight gain, feed intake, and occluding mRNA expression, and increased feed conversion ratio, serum endotoxin, and pro-inflammatory markers (Chen et al., 2015).

Systemic inflammation in poultry has nutritional and energetic costs, which in turn create economic costs – the cost of prophylaxis, the cost of treatment, and the cost of lost performance. To mitigate these losses, the industry has explored a wide range of alternatives to AGPs. However, no single alternative has yet proven as effective as AGP against the broad spectrum of bacterial challenges. Successful approaches to AGP-free production generally involve the use of multiple alternative products, each having different modes of action. Therefore, strategic use of AGP alternatives requires both an understanding of the modes of action and potential targets of each product and the likely bacterial challenges of concern in each particular operation.

## The role of fiber in gut inflammation (R. Jha)

Gut inflammation is a common occurrence in poultry, which is triggered by various factors, including pathogens, stress, and dietary imbalances. Gut inflammation can lead to intestinal barrier dysfunction, impaired nutrient absorption, and a reduction in overall performance. Among several nutritional strategies, the inclusion of dietary fiber has been proposed as a means to address this issue, with notable success. Dietary fiber was once considered an anti-nutritional factor. However, it is now recognized for its potential for functional benefits in modulating gut health and alleviating inflammation (Jha and Mishra, 2021).

Dietary fiber includes a diverse group of polysaccharides and lignin that are resistant to digestion by endogenous enzymes in the small intestine. It is broadly classified based on its physicochemical properties, such as solubility, fermentability, and viscosity. Insoluble fibers primarily contribute to gut motility and digesta viscosity, whereas soluble fibers are readily fermented by gut microbiota, producing functional metabolites (Jha and Berrocoso, 2015). The specific effects of fiber on gut inflammation depend on its physicochemical properties and interaction with the gut environment.

Dietary fiber helps manage gut inflammation through various physical and physiological mechanisms. Insoluble fibers increase digesta viscosity and gut motility, thereby influencing gut transit time and nutrient absorption. However, the reduced passage rate of digesta allows the harmful bacteria to flourish on undigested nutrients. Fiber can also physically bind to pathogens, reducing their adherence to the intestinal mucosa and mitigating their pathogenic effects (Jha and Mishra, 2021). Furthermore, fiber can influence gut morphology by increasing villus height and crypt depth, enhancing the absorptive surface area (Singh et al., 2021). Also, certain fibers serve as substrates for beneficial gut bacteria, which produce short-chain fatty acids (SCFAs) like acetate, propionate, and butyrate. SCFAs provide energy for enterocytes and promote proliferation and differentiation of cells in the villi and crypts. Butyrate, in particular, serves as a primary energy source for colonocytes and plays a crucial role in maintaining gut barrier integrity. SCFAs exert anti-inflammatory effects by inhibiting the NF-κB signaling pathway, reducing pro-inflammatory cytokine production, and enhancing expression of tight junction proteins, such as occludin and claudin, and stimulating mucus production (Jha and Mishra, 2021; Liu et al., 2023). However, excessive fiber inclusion can mechanically damage intestinal villi, leading to impaired nutrient absorption and increased inflammatory responses (Jha and Berrocoso, 2016). Moreover, specific dietary fibers serve as a prebiotic, thereby selectively promoting the growth of beneficial bacteria, such as Lactobacillus and Bifidobacterium, while inhibiting the growth of pathogenic bacteria, such as *Clostridium perfringens* (Yadav and Jha, 2019). This modulation of the gut microbiota can enhance gut barrier function and immune responses via various mechanisms such as increasing mucus production, stimulating epithelial cell proliferation, producing SCFAs, and supporting tight junction proteins (TJPs). These, in turn, contribute to reducing inflammation. Dietary fiber influences immune responses by altering cytokine production and immune cell activity. Microbial fermentation products of dietary fiber, SCFAs, interact with immune cells to reduce pro-inflammatory cytokines, such as IL-6, thereby alleviating gut inflammation (Jha et al., 2019). However, non-fermentable fibers may trigger immune activation due to their abrasive nature on the gut lining (Jha and Berrocoso, 2015).

For practical considerations, the optimal fiber inclusion levels in poultry diets depend on various factors, including type, age, and production stage of poultry. Fiber sources should be selected for their prebiotic functions to support the beneficial microbiota. Excessive fiber can negatively impact nutrient digestibility and growth performance, while insufficient fiber may compromise gut health. However, fiber-degrading enzymes can be used to mitigate their adverse effects (Singh et al., 2021). Also, the interactions between fiber and other dietary components, such as proteins and minerals, must be considered when managing gut health (Jha and Berrocoso, 2016). It is because fiber does not function in isolation within the digestive system. Different fiber fractions, such as soluble and insoluble fibers, can change how nutrients are digested, absorbed, or fermented by the gut microbiota. Also, dietary fiber supplementation can be a valuable strategy to mitigate gut inflammation challenges, such as necrotic enteritis and coccidiosis (Adhikari et al., 2020). Moreover, use of prebiotics and enzymes in conjunction with dietary fiber may further enhance overall gut health (Singh et al., 2021; Mishra et al., 2024).

Future research should focus on describing the specific mechanisms by which different fiber types and sources modulate gut inflammation in poultry. Additionally, further investigation is needed into the impact of fiber processing on its physiological effects. The potential of using novel fiber sources, including fibrous feedstuffs and prebiotics, to enhance gut health should be explored. More studies are needed to investigate the interactions between fiber, gut microbiota, and immune function, which play crucial roles in managing gut inflammation. Meta-analyses of existing research can provide valuable insights into the optimal use of fiber in poultry diets.

In conclusion, dietary fiber plays a multifaceted role in modulating gut inflammation in poultry through multiple mechanisms, including physical effects, SCFA production, gut barrier enhancement, microbiota modulation, and immune regulation. While moderate levels of fermentable fiber promote gut integrity and immune homeostasis, excessive or inappropriate fiber sources can exacerbate gut inflammation. Thus, optimizing fiber inclusion in poultry diets can contribute to improved gut health, enhanced performance, and reduced disease susceptibility. Further research is needed to understand the complex interplay between fiber and gut health and to develop strategies for its optimal utilization in poultry.

## It’s not just about digestibility: the role of amino acids in inflammation (W. Kim)

Enteric diseases and environmental stress are important economic and health issues for the poultry industry (Castro et al., 2020a; Teng et al., 2023). Enteric diseases, such as coccidiosis and necrotic enteritis, damage the intestine and cause inflammation and oxidative stress, considerably affecting growth performance, gut and bone health, and overall health of poultry (Teng et al., 2020; Tompkins et al., 2023; Goo et al., 2024b). Minimizing inflammation and oxidative stress during infection periods is critical to alleviating the detrimental effects and maintaining efficient gut and skeletal integrity and organ development in poultry. Therefore, identifying nutritional strategies to mitigate the negative effects of enteric diseases in poultry is critical.

Amino acids are important building blocks of muscle protein synthesis (Wu, 2009; Castro and Kim, 2020a). Beyond their role in muscle growth, certain amino acids have functional roles that may directly or indirectly manage inflammation and oxidative stress caused by enteric diseases (Castro et al., 2020b; Kim et al., 2022; Liu and Kim, 2023). There are several amino acids with functional roles in poultry: arginine, methionine, threonine, glutamine, and branched-chain amino acids (BCAA: Leucine, Valine, and Isoleucine). First, methionine is an essential amino acid, which is important for muscle accretion and DNA methylation, and is a precursor of glutathione and taurine, which have anti-inflammatory and antioxidant properties in the body (Stipanuk, 2004). In addition, the metabolite of methionine, S-adenosyl-l-methionine enhances antioxidant enzymes, superoxide dismutase, and catalase, to neutralize oxidative stress (Lozano-Sepulveda et al., 2016). It has been shown that methionine supplementation enhanced TJP expression, intestinal development, and antioxidant status in broilers and reduced inflammation and oxidative stress (Miao et al., 2021). Methionine to cysteine ratio (MCR) in the diet is also important to maintain efficient growth, immunity, intestinal health, and oxidative status in broilers under enteric disease conditions. As MCR decreased, growth was retarded, serum total antioxidant capacity was linearly suppressed, and the activities of hepatic glutathione peroxidase and superoxide dismutase were quadratically reduced in broilers affected by coccidiosis (Liu et al., 2024).

Threonine also has functional properties. Under enteric disease conditions, threonine requirements may be elevated to maintain gut integrity and functionality by promoting mucin secretion from goblet cells (Faure et al., 2002). Threonine supplementation improved body weight gain and feed intake in broilers under necrotic enteritis conditions (Star et al., 2012). Glutamine plays a role in maintaining gut health and controlling inflammation under enteric disease conditions; it can be utilized as an energy source for enterocytes and alleviates inflammation by reducing pro-inflammatory cytokines (Bortoluzzi et al., 2018; Teng et al., 2021). In broilers under cocci vaccine challenge, 0.5-1 % glutamine supplementation improved TJP expression, intestinal morphology, and growth performance and reduced pro-inflammatory cytokine production (Oxford and Selvaraj, 2019).

BCAAs are essential amino acids, and maintaining their proper balance in diets is critical for optimal production and overall health (Goo et al., 2024a). BCAAs also have antioxidant and anti-inflammatory properties to improve gut health and maintain efficient growth in poultry (Kim et al., 2022). Additional BCAAs supplementation improved growth performance and intestinal integrity in broilers challenged with mixed Eimeria spp. (Liu et al., 2023; Ajao et al., 2024). Moreover, valine and isoleucine deficiency significantly suppressed growth performance, muscle accretion, and intestinal health in broilers challenged with necrotic enteritis (Goo et al., 2025), indicating that adequate supplementation of valine and isoleucine is critical for maintaining efficient growth and a healthy gut environment in broilers facing enteric disease challenges.

Arginine is a multifunctional amino acid, which is involved in protein synthesis, nitric oxide production, antioxidant mechanism, anti-inflammatory process, angiogenesis, proline production, and polyamine production (Castro and Kim, 2020; Kim et al., 2022). It has been demonstrated that arginine inhibits IL-1B-induced NF-kB pathway, which is important for inflammation (Meng et al., 2017); arginine interrupts NF-kB binding activity on the promoters of inflammatory genes, reducing inflammation. Thus, arginine has been shown to have beneficial effects on growth performance and gut health in poultry under enteric disease and environmental stress conditions. Arginine supplementation significantly improved growth performance and TJP expression and reduced gut permeability in broilers infected with coccidiosis (Castro et al., 2020b; Teng et al., 2021; Liu et al., 2023), indicating that functional properties of arginine help minimize intestinal damage and stimulate recovery in broilers under enteric disease conditions.

Another nutritional strategy to alleviate the inflammation and severity caused by enteric diseases is lowering crude protein and supplementing balanced amino acids or functional amino acids in poultry diets. It has been demonstrated that higher dietary protein levels increase intestinal damage and inflammation under disease conditions (Bryan et al., 2019; Sung and Adeola, 2025). In addition, functional amino acid supplementation in low protein diets with balanced essential amino acids alleviated inflammation and detrimental effects under coccidiosis conditions (Teng et al., 2021; Liu et al., 2023; Taylor et al., 2024), indicating that supplementing balanced amino acids and functional amino acids in low protein diets is a potential nutritional strategy to modulate inflammation and improve gut health in broilers under enteric disease conditions.

Strategic use of such nutritional approaches and understanding of the mode of actions of specific functional amino acids in poultry under enteric disease conditions will maintain efficient production and improve gut health in poultry, promoting sustainable poultry production.

## Mineral chelation with phytate and its impact on nutritional immunity and gut inflammation (L. Marchal and K. Gibbs)

Trace minerals (**TM**) are essential for metabolism: Fe supports oxygen and electron transport and DNA synthesis; Cu participates in key metabolic pathways, including detoxification; Zn regulates carbohydrate metabolism and protein synthesis; and Mn is vital for lipid metabolism, bone, and eggshell formation (Suttle, 2010). Optimal levels of TM in the diet also support the functioning of beneficial intestinal microflora, thereby enhancing gut health and barrier integrity, whereas excessive levels of individual TM can be harmful, for example, excessive Zn inhibits absorption and utilization of Cu and Fe (Bao et al., 2010), and alters gut morphology (Wang et al., 2023), impairing nutrition. Birds employ dynamic and highly evolved mechanisms to regulate TM bioavailability depending on the immediate physiological need, either sequestering minerals to restrict their use by pathogens for growth, or concentrating them in immune cells to kill bacteria, a process known as nutritional immunity (Hood and Skaar, 2012). Supplying the correct level of TM in the diet to support this balance is key.

Feed raw materials contain TM in relative abundance, but their bioavailability is variable due to chelation by plant-derived phytate at intestinal pH (Maenz et al., 1999). Phytate forms binary and ternary complexes with TM depending on the pH and the presence of other cations or ligands (such as protein; Selle et al., 2012). These complexes are generally insoluble and poorly absorbed in the gut. Phytate can therefore reduce the bioavailability of TM, which is important for maintaining gut epithelial structure and barrier integrity (e.g., Zn), or can also promote intestinal inflammation due to excess mucin production to counteract phytate irritation (Selle et al., 2012). To avoid dietary TM availability being insufficient, Fe, Zn, Cu, Mn, and Se are added to feed, but often at levels far more than actual requirements (Franklin et al., 2022). As well as enabling pathogen growth, excess TM can adversely impact the bird microbiome. When excreted, TM can affect soil, water, and crop ecosystems and accumulate in the food chain.

The antinutritive effects of phytate in feed can be reduced by supplementation with exogenous phytase that hydrolyzes phytate to release inorganic phosphate (**iP**) and lower inositol phosphate esters. A recent study has shown that exogenous phytase can fully replace supplemental TM (Zn, Fe, Cu and Mn) in broiler diets, regaining growth performance and maintaining or improving tissue TM concentrations similar to those in birds fed diets with conventional TM supplementation (Dersjant-Li et al., 2025). The effect was attributed to the capacity of the phytase to extensively hydrolyze phytate at the low pH conditions of the upper gastrointestinal tract (Christensen et al., 2020; Dersjant-Li et al., 2022b), thus reducing phytate-TM binding and increasing the bioavailability of native TM in the diet. These findings prompt questions about whether TM supplementation could be reduced in broiler diets containing phytase to improve feed efficiency and reduce TM waste. However, such dietary changes could also have implications for two related concepts: nutritional virulence and nutritional immunity. Nutritional virulence refers to the ability of pathogens to exploit host nutritional resources (such as unbound dietary TM) to support their own persistence and pathogenicity. By reducing excess dietary TM, nutritional virulence may be suppressed. Likewise, minimizing excess TM could also enhance nutritional immunity, defined as the host’s capacity to restrict the availability of essential nutrients to the pathogen, thereby limiting pathogen growth and survival. Existing studies have shown that directly reducing TM supplementation (even without phytase supplementation) enhances nutritional immunity by restricting nutrient availability to pathogens (Hood and Skaar, 2012; Trairatapiwan et al., 2025). The remaining question is whether feed additives such as phytase, in combination with TM reduction, could enhance this effect?

In theory, the combination of supplemental phytase with low (reduced) TM inclusion in the diet could reduce nutritional virulence and enhance nutritional immunity via a number of different routes. First, formulating diets to more precisely meet TM requirements might better meet the physiological needs of the bird by enabling birds to more effectively sequester remaining circulating concentrations of TM, such as Fe and Zn, during immune responses and periods of inflammation (Hennigar and McClung, 2016). Second, it might reduce opportunities for sequestration of TM by pathogens themselves, thereby reducing their growth and persistence (nutritional virulence). Avian pathogenic *Escherichia coli* (**APEC**) that causes economically relevant diseases in poultry, such as colibacillosis, is one opportunistic target to consider here. Several evolutionary pathways could explain how APEC acquired pathogenicity (Mageiros et al., 2021), but all have resulted in the acquisition of virulence-associated genes, including those enabling the sequestration of host nutrients, in particular Fe. Iron is sequestered by APEC via siderophores (specialized small molecules secreted by the bacteria), which bind ferric Fe. In defense, the host secretes proteins that bind siderophores, blocking APEC access to Fe (Wilson et al., 2016). Reducing excess Fe in the GIT by phytase supplementation and reducing Fe supplementation might assist with reducing APEC nutritional virulence. However, avoiding deficiency is also important, since Fe is essential for the development and functioning of immune cells; deficiency impairs gut morphology and function and alters the microbiome composition (Chen et al., 2025).

Iron is also a nutritional virulence factor of the food safety pathogen *Campylobacter*, alongside iP, which has been recognized as a contributor to *Campylobacter* virulence and pathogenesis (Sinha et al., 2020). Campylobacteriosis (most commonly caused by *C. jejuni*) is among the top four global causes of human gastroenteritis, and 50–80 % of cases originate from poultry (Kelbert et al., 2025). No single control strategy is fully effective, and producers must rely on combining multiple interventions and adapting strategies to specific scenarios. Birds have evolved mechanism to limit free Fe in the gastrointestinal tract (**GIT**) during a *Campylobacter* infection by chelating it to transferrins such as ovotransferrin (Chan et al., 2023). However, *Campylobacter* has adapted to exploit host-chelated Fe by scavenging siderophores produced by other bacteria. A recent, small-scale, in-vivo study (Gibbs et al., 2025) on the impact of removal of added dietary Fe and iP in combination with phytase supplementation in *Campylobacter*-challenged broilers identified a 0.88 Log reduction in *Campylobacter* colony forming units per gram of ceca on d 42, following an oral challenge on d 14. In addition, there was a 1 Log10 unit reduction in the upper end of the range of *Campylobacter* cecal loads in the phytase-supplemented birds. These preliminary findings suggest that the combination of Fe reduction and phytase supplementation reduced the capacity of *Campylobacter* to colonize the GIT. As phytase is already used almost ubiquitously, this small dietary modification of reducing added Fe with phytase could form part of an intervention in practical diets.

TM bioavailability has a significant impact on bird nutrition as well as on gut health, immune functioning, and the ability to respond effectively to pathogen challenge. Evidence supports that using a high dose of an effective phytase can make phytate-bound TM in the feed raw material bioavailable for the bird, reducing the need to add TM to the diet. So, a high dose of phytase supplementation can play an important role in achieving fed diets that are optimized in TM content. The potential impact of this resonates further than nutrition, because optimized formulation also supports immune mechanisms and reduces the risk of opportunistic microbial behavior. Further research is warranted in this field.

## Managing subclinical inflammation from a commercial perspective- a nutritionist's point of view (J. Halley)

Interest in the use of subclinical infection as a means of identifying lack of performance in growing broilers began to take off in the 1980′s. Researchers such as Kirk Klasing began publishing their work showing the effect of challenge or nutrient deficit on the immune system and its resulting increase in the nutrient deficit. It was not hard to imagine the challenges that a broiler chicken faced in those days from their environment, as well as diseases and other influences. Dr. Robert Teeter, working at Oklahoma State University, and his colleagues investigated the relationship between various environmental and husbandry practices and their effect on energetic efficiency.

While there is an abundance of literature that clearly shows the positive effect that nutrients such as Vitamin D, Zinc, Copper, and Selenium can have on the immune system of broiler chickens, there is also an abundance of anecdotal observations made by poultry professionals on these nutrients during various disease situations where there is an almost “pharmaceutical” response. Various countries around the world have different maximum use rates for some of these nutrients in feed, and quite often, very positive effects are seen when they are used in levels higher than allowed in Europe and the United States.

The hatching system has such a profound effect on the health and productivity of the chicks that not enough can be said or done to make sure that the hatchery is operating within proper parameters at all times. Because of the different environmental temperatures, humidity, etc. that hatcheries have to contend with year-round it is critical that the ability to maintain the proper hatching parameters is critical, as this is the start of the whole production cycle; if it starts badly, it will not improve.

Critical to maintaining health status is the quality and availability of fresh water. It is often taken for granted, and the routine cleaning, flushing, and filtering of the drinking water is often neglected, resulting in compromised intakes and reduced body weight gains. More attention paid to this area will always result in improvements in production and economic returns. The biofilm in water lines is populated predominantly by Salmonella and Campylobacter species, all unwanted bacteria that can be controlled through proper sanitation conducted on a routine basis.

The following ingredients have been shown in research trials to help modulate the immune system of broiler chickens:

Vitamin D – At levels above its nutritional use, it stimulates the immune system, whereas dietary deficiency of vitamin D reduces immune function (Aslam et al., 1998). Vitamin E and Se – Supplementing these nutrients helps to maintain the heterophil/lymphocyte ratio during inflammation and increases an antigen-specific response following immunization. Deficiency of vitamin E reduces immune capability (Erf et al., 1998). Vitamin A deficiency may result in reduced cellular immunity responses. Excess increases humoral immunity, which may impair the antibody response. Fat – The fatty acid profile, saturation, and carbon bond lengths all affect the formation of signaling compounds called eicosanoids, which are involved in the immune response. Protein (Amino Acid) imbalance or deficiency: Methionine is first limiting in corn/soy diets, and birds that are challenged with an infection, especially of the gut, need more Methionine and other amino acids such as Threonine, Glutamine, and Arginine to overcome this challenge. Deficiency of protein in the diet has been shown to reduce immune function (Jahanian, 2009), as well as deficiencies of individual amino acids, lysine (Chen, et al., 2003), arginine (Jahanian, 2009), and methionine (Kanashi, et al., 2000; Zang and Guo, 2008).

## CRediT authorship contribution statement

**Rajesh Jha:** Writing – review & editing, Writing – original draft, Conceptualization. **Doug Korver:** Writing – review & editing, Writing – original draft, Conceptualization. **Woo Kyun Kim:** Writing – review & editing, Writing – original draft, Conceptualization. **Leon Marchal:** Writing – review & editing, Writing – original draft, Conceptualization. **Kirsty Gibbs:** Writing – review & editing, Writing – original draft, Conceptualization. **John Halley:** Writing – review & editing, Writing – original draft, Conceptualization.

## Disclosures

Leon Marchal and Kirsty Gibbs are employed by the Danisco Animal Nutrition & Health, IFF, Oegstgeest, the Netherlands, which sponsored the symposium. All authors declare that they have no known competing financial interests or personal relationships that could have influenced the work reported in this paper.
